# Inflammatory Response in the Anterior Chamber after Implantation of an Angle-Supported Lens in Phakic Myopic Eyes

**DOI:** 10.1155/2014/923691

**Published:** 2014-05-25

**Authors:** Suphi Taneri, Saskia Oehler, Carsten Heinz

**Affiliations:** Center for Refractive Surgery, Eye Department, St. Francis Hospital, Hohenzollernring 57, 48145 Muenster, Germany

## Abstract

*Purpose*. To evaluate the inflammatory reaction after implantation of an angle-supported foldable acrylic anterior chamber IOL for myopia correction over time. *Methods*. Adult individuals seeking vision correction with stable myopia >7.0 D were included. Exclusion criteria are anterior chamber depth <2.8 mm, insufficient endothelial cell density, other preexisting ocular conditions, and prior eye surgery. Laser flare photometry and slitlamp examination were performed before and up to 1 year after implantation of an AcrySof Cachet IOL (Alcon Laboratories, Forth Worth, TX, USA). Postoperative treatment comprised antibiotic eye-drops for 5 days and nonsteroidal anti-inflammatory eye-drops (NSAIDs) for 4 weeks. *Results*. Average laser flare values of 15 consecutive eyes of 15 patients were 8.3 ± 9.7 preoperatively and 19.0 ± 24.2 (1 day), 24.0 ± 27.5 (1 week), 17.6 ± 13.4 (1 month), 14.9 ± 15.4 (3 months), and 10.0 ± 7.0 (1 year) photon counts/ms after implantation, respectively. Slitlamp examination yielded 0 or 1+ cells (SUN classification) in every one eye throughout the follow-up period. *Conclusion*. Results indicate a low maximum inflammatory response and a quick recovery to a long-term safe level. The use of NSAIDs seems sufficient in routine cases, thus avoiding potential drawbacks of using corticoids.

## 1. Introduction

Complications utilizing several types of phakic intraocular lenses (pIOLs) to correct refractive errors are rare [1]. However, some concerns about biocompatibility in the long term remain and need to be evaluated individually for every implant model [2–5].

**Figure 1:**
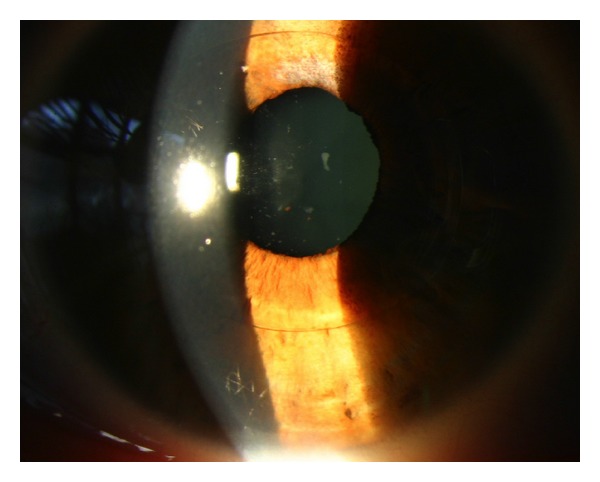
Foldable iris claw fixated anterior chamber IOL made from hydrophobic polysiloxane 2 months after implantation showing typical inflammatory deposits despite continuous local fluorometholone t.i.d. medication.

Delicate intraocular structures including the corneal endothelium, iris, crystalline lens, and the anterior chamber angle may be altered by inflammatory processes after lens implantation. Some types of anterior chamber IOLs made from silicone require the use of steroidal eye-drops for 2-3 months after implantation to control the inflammatory response (Figure 1).

A newly developed foldable anterior chamber angle-supported IOL is called AcrySof Cachet (Alcon Laboratories, Inc., Fort Worth, USA). This model is CE-marked and currently undergoing US Food and Drug Administration (FDA) clinical investigation for the correction of moderate to high myopia [6, 7].

We wanted to assess anterior chamber reaction after implantation of the AcrySof Cachet in phakic myopic eyes by laser flare photometry in addition to slitlamp examination over time to evaluate its biocompatibility in a clinical setting.

## 2. Patients and Methods

This prospective case series included healthy eyes of Caucasian patients with stable myopia >7.0 D in which an AcrySof Cachet pIOL (Alcon Laboratories, Fort Worth, USA) was implanted to minimize refractive error. No eye was eligible for full correction with an excimer laser. Exclusion criteria were anterior chamber depth <3.2 mm (measured including corneal thickness), insufficient endothelial cell density according to manufacturer's age-related recommendations, other preexisting ocular conditions, and prior eye surgery. Patient demographics and preoperative refraction are summarized in Table 1. Approval from the local ethics committee (University of Muenster) was obtained. All patients were informed and gave their written consent. The study was performed according to the ethical principles of the Declaration of Helsinki. Fifteen eyes of 15 consecutive patients were evaluated preoperatively and at the follow-up visits scheduled 1 day, 1 week, 1 month, 3 months, and 1 year after implantation, respectively.

### 2.1. Preoperative Examinations

Prior to pIOL implantation, patients had a complete ophthalmologic evaluation, including subjective refraction, slitlamp exam, Goldmann applanation tonometry, and binocular indirect ophthalmoscopy through dilated pupils. Objective refraction was obtained with the Canon R-F10 (Canon, Tokyo, Japan). Keratometry, axial length, and anterior chamber depth were obtained with the IOL Master (Carl Zeiss Meditec AG, Jena, Germany). The endothelium (including cell density) was evaluated using a specular microscope (SP-3000 P, Topcon, Willich, Germany). The horizontal corneal diameter was determined using the IOL Master and the Orbscan IIz (Technolas Perfect Vision, Munich, Germany) to choose the size of the implant. Laser flare photometry was obtained using Kowa FM-500 (Kowa Company Ltd., Tokyo, Japan).

### 2.2. Surgical Procedure and Postoperative Treatment

All surgery was performed under general anesthesia by the same experienced surgeon (ST). Prior to surgery, several drops of pilocarpine 0.5% were administered to ensure pupil constriction. A square corneal incision using a 2.5 mm blade was made. A cohesive viscoelastic device (ProVisc, Alcon Laboratories, Fort Worth, USA) was injected to maintain anterior chamber depth. The AcrySof Cachet was then implanted using the Monarch III (Alcon Laboratories) injector with a P cartridge. The viscoelastic device was removed by manual irrigation through the tunnel. No further medication was administered intracamerally. Topical antibiotics were given and a patch was applied for three hours. Then a slitlamp exam including applanation tonometry was performed.

Postoperative treatment was limited to unpreserved ofloxacin eye-drops 4 q.i.d. for 5 days (Floxal EDO, Dr. Mann Pharma GmbH, Berlin Germany) and unpreserved flurbiprofen eye-drops (Ocuflur O.K., Pharm-Allergan, Ettlingen, Germany) q.i.d. for 4 weeks.

### 2.3. Assessment of the Inflammatory Response

**Figure 2:**
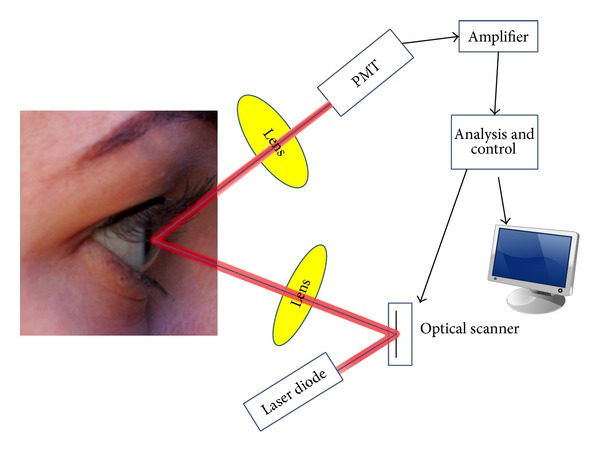
Measurement principle of laser flare photometry based on laser light scattering detection. A measurement window is projected inside the anterior chamber of the eye and is scanned by a diode laser beam. As an aqueous protein or another particle (i.e., component of inflammation) passes through the focal point of the laser, light scattering occurs. A photomultiplier-tube then detects the intensity of the scattered light, which is directly proportional to the amount of protein particles or flare, and generates an electrical signal. These signals are processed by a computer and immediately displayed for user analysis.

The inflammatory response of the anterior chamber was assessed according to the standardization of uveitis nomenclature (SUN) working group by the same experienced examiner [8]. In addition, laser flare photometry was used as an objective method. Laser flare photometry is based on the measurement principle of laser light scattering detection. A measurement window is projected inside the anterior chamber of the eye and is scanned by a diode laser beam. As an aqueous protein or another particle (i.e., component of inflammation) passes through the focal point of the laser, light scattering occurs. A photomultiplier-tube then detects the intensity of the scattered light, which is directly proportional to the amount of protein particles or flare, and generates an electrical signal. These signals are processed by a computer and immediately displayed for user analysis (Figure 2). Measurement results are given in photon counts per millisecond (pc/ms).

Laser flare counts were performed before applanation tonometry or administering any eye-drops to avoid artifacts. Ten laser flare measurements per eye were carried out by experienced technicians. Of these, the highest and the lowest values were discarded and the remaining eight sequential scans were then averaged to obtain a flare count for this individual eye according to the recommendations put forth by the Kowa Laser Flare-Cell Photometry Medical Advisory Board [9]. If the standard deviation of these remaining eight readings was higher than 10 pc/ms, the measurement was repeated.

### 2.4. Statistical Analysis

The arithmetic mean, the standard deviation, and the upper and lower quartiles of the flare count for the whole cohort were calculated for each point in time.

To test for Gaussian distribution of the laser flare values, a nonparametric test (Kolmogorov-Smirnov) was used. To test for statistical differences of the scaled laser flare counts, the Wilcoxon rank sum test was used. A *P* value less than 0.025 was considered statistically significant. Statistical analysis was performed using SPSS for Mac software (version 20, SPSS Inc.) and Excel 2008 for Mac software (version 12.1.0, Microsoft Corp.).

## 3. Results

No complication (e.g., intraocular pressure spikes, dislocation of the phakic IOL, or cataract induction, etc.) occurred intraoperatively and during the follow-up period. Postoperative visual outcomes are provided in Table 2 and pre- and postoperative endothelial cell density are provided in Table 3.

**Figure 3:**
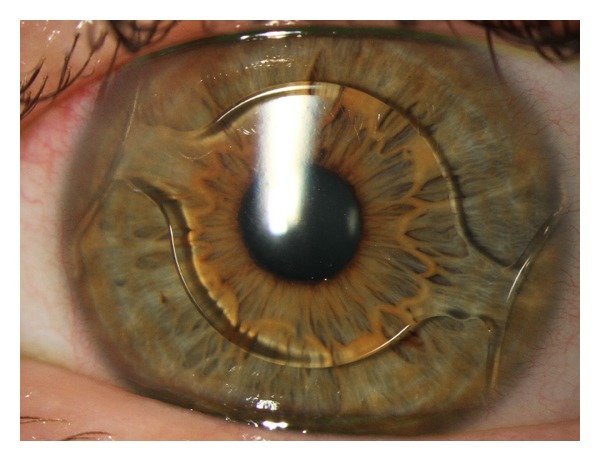
Typical slitlamp findings on the first day after implantation of an AcrySof Cachet showing minimal inflammatory response. Note the conjunctival injection at the corneoscleral tunnel at 10 o'clock.

Slitlamp evaluations of the anterior chamber revealed 0 to 10 cells within the measurement window (1 mm × 1 mm) in every one eye at all preoperative exams and 1 day and 1 week follow-up visits, respectively (Figure 3). This corresponds to “0” to “1+” according to the SUN working group classification [8]. No cells were found at 1 month and thereafter. No aqueous flare was noted in any eye.

**Figure 4:**
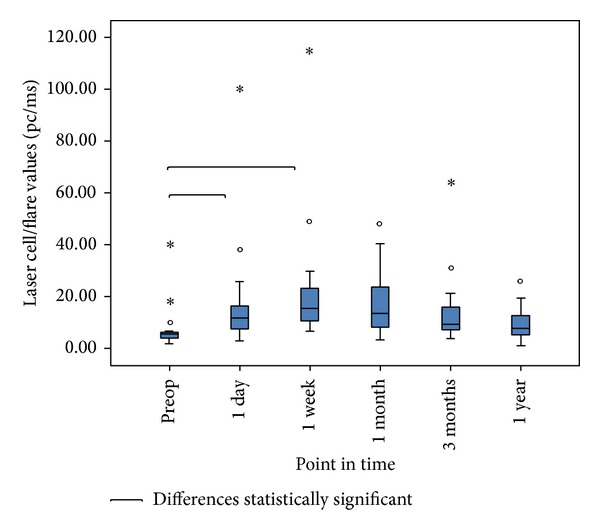
Box plot of laser flare values over time. Gray boxes span the 1st quartile to the 3rd quartile, including the median. Whiskers denote the lower and higher extreme within the 1.5 interquartile range. Circles denote mild outliers; asterisks denote extreme outliers. Square brackets denote statistical significant increases of laser flare values compared to baseline.

Preoperative laser flare values of 15 consecutive eyes were 8.3 ± 9.7 (range 1.8–40.4) pc/ms. After lens implantation, the measurements were more challenging and had sometimes to be repeated to obtain readings with an acceptable standard deviation. Postoperative laser flare values were elevated to 19.0 ± 24.2 (range 3.1–100.3) pc/ms at day 1 and to 24.0 ± 27.5 (range 6.6–114.9) pc/ms after one week. One month postoperatively, laser flare values had declined to 17.6 ± 13.4 (range 3.4–48.6) pc/ms. Three months and one year after implantation, laser flare values were 14.9 ± 15.4 (range 3.8–63.9) pc/ms and 10.0 ± 7.0 (range 1.1–26.0) pc/ms, respectively. Laser flare values are summarized in Figure 4. Statistical significant increases of laser flare values compared to baseline were observed 1 day and 1 week after implantation but not after 1 month, 3 months, and one year.

## 4. Discussion

To the best of our knowledge, this is the first study examining the inflammatory response after implantation of an AcrySof Cachet pIOL. By using laser flare photometry in addition to slitlamp evaluation, we wanted to provide objective data. Laser flare photometry is the current standard method in studies to quantify inflammation and disturbance of the blood-aqueous barrier following surgery with good accuracy and reproducibility. It is capable of detecting very slight increases in flare values undetectable by conventional slitlamp examination and thus is the preferred method for detecting subclinical inflammation processes [9–12].

With both methods, we found a relatively low initial reaction and a quick recovery to normal values without using steroids in the postoperative regimen.

However, our study is limited by the size of the cohort and the followup of 1 year.

A further limitation is that we did not compare postoperative inflammation with and without the application of steroids.

Comparing our results with previously published data, we found that the preoperative mean laser flare value in our study is in good agreement with values determined in other healthy eyes of this age group [13]. The increased difficulty of laser flare measurements with an implant in the anterior chamber is reflecting in the markedly increased standard deviation of postoperative readings compared to the standard deviation of preoperative readings. As we repeated measurements to obtain readings with an acceptable standard deviation for a single eye, we assume that the high standard deviation for all eyes results from individual differences of the eyes and not from invalid measurements. We may speculate that these different responses may largely be due to varying amount of viscoelastic devices inadvertently retained in the anterior chamber at the end of surgery.

One day after pIOL implantation, the mean laser flare value increased and peaked after one week at approximately 25 pc/ms in our cohort. On the background that in uveitic eyes levels of 300 pc/ms or more may be reached, this initial reaction to the surgical trauma may be regarded as “low grade inflammation.” One month after surgery, the laser flare value had decreased and anti-inflammatory medication was discontinued. Noteworthy, commonly recommended steroids were not used additionally in this study. Several studies have shown the efficacy of NSAIDs in the postoperative treatment of cataract surgery [14, 15]. We found only one study that evaluated topical bromfenac sodium versus betamethasone sodium after implantation of the Artisan and the Artiflex pIOLs (Ophtec BV, Groningen, The Netherlands) and observed that both are equally effective for controlling postoperative inflammation [16]. Given the potential side effects of local steroids, including a rise in intraocular pressure and the formation of cataracts [17], we feel that the application of NSAIDs alone might be preferable to the combined use with steroids after uneventful implantation of pIOLs in healthy eyes of young adults. However, this should be verified in larger case series.

Laser flare values in our patients remained almost stable at 3, 6, and 12 months postoperatively at a level that may be regarded as safe in the long term.

There are only a few studies evaluating the uveal biocompatibility using laser flare photometry after implantation of an anterior chamber pIOL. In extensively studied uveitic eyes, chronic laser flare counts >20 pc/ms are associated with an increased rate of complications [18]. Pérez-Santonja et al. found a low grade subclinical inflammation with constantly elevated laser flare values up to 2 years after implantation of an iris-fixated and an angle-supported anterior chamber pIOL [2, 3]. In comparison to that, we found a lower maximum inflammatory response in the anterior chamber and a quicker recovery. Yamaguchi et al. measured lower absolute laser flare values than we did after implantation of an iris-fixated pIOL. However, as no preoperative values are provided, assessing the inflammation in relation to the respective baseline of the different cohorts is impossible [5].

When using iris-fixated pIOL, a rupture of the blood-aqueous barrier and subsequent subclinical inflammation are attributed to the incarceration of the iris by the lobster claw [19]. Utilizing angle-supported lenses, it had been speculated that a prolonged compression of the haptics against uveal tissue might induce a certain amount of anterior inflammation [20]. However, our results indicate a low inflammatory response for an angle-supported pIOL. This might be attributable to the less traumatic implantation (without damage to the iris) in the short term as well as the material of this lens in the long term. The AcrySof Cachet is composed of a hydrophobic acrylate, which has a long track record of excellent biocompatibility [21, 22]. Moreover, the haptic design may also play a crucial role.

In summary, our results indicate a low maximum inflammatory response after implantation of an AcrySof Cachet, a quick recovery to safe values at 1 month without using steroids and stable values up to one year that are comparable to preoperative values. Larger studies with followups of several years are warranted.

## Figures and Tables

**Table 1:** Patient demographics and preoperative refraction.

Parameter	Result
Age (y)	
Median	38
Range	21 to 59
Corrected distance visual acuity (decimal)	
Mean	0.68
Range	0.40 to 1.00
Refraction (D)	
Mean MRSE ± SD	−11.25 ± 3.34
Range	−20.50 to −5.88
Mean sphere ± SD	−10.52 ± 3.27
Range	−19.50 to −5.00
Mean cylinder ± SD	−1.49 ± 0.92
Range	−3.75 to 0.00

**Table 2:** Postoperative visual outcomes.

Parameter	Result
Uncorrected distance visual acuity (decimal)	
1 day	
Mean	0.70
Range	0.30 to 1.25
1 week	
Mean	0.77
Range	0.50 to 1.25
1 month	
Mean	0.86
Range	0.60 to 1.25
3 months	
Mean	0.87
Range	0.60 to 1.25
1 year	
Mean	0.86
Range	0.40 to 1.25

Refraction (D)	
3 months	
Mean MRSE ± SD	−0.45 ± 0.55
Range	−1.50 to 0.25
Mean sphere ± SD	0.03 ± 0.57
Range	−1.25 to 0.75
Mean cylinder ± SD	−0.97 ± 0.54
Range	−1.75 to −0.25
1 year	
Mean MRSE ± SD	−0.46 ± 0.51
Range	−1.38 to 0.63
Mean sphere ± SD	−0.11 ± 0.57
Range	−1.25 to 1.00
Mean cylinder ± SD	−0.70 ± 0.67
Range	−2.50 to 0.00

**Table 3:** Endothelial cell density.

Parameter	Result
Preoperative endothelial cell density (cells/mm^2^)	
Mean	2675.9 ± 311.0
Range	2217.1 to 3086.7
Postoperative endothelial cell density (cells/mm^2^)	
1 month	
Mean	2607.8 ± 319.0
Range	2221.6 to 3201.4
3 months	
Mean	2509.5 ± 392.8
Range	1931.0 to 3144.5
1 year	
Mean	2825.0 ± 216.0
Range	2520.1 to 3217.4

## What Was Known

- Several phakic IOL types have been used to correct moderate-to-high myopia in adult patients. However, some concerns about potential long-term risks to anterior segment structures due to (subclinical) anterior chamber inflammation after pIOL implantation still remain.

## What This Paper Adds

- Anterior chamber inflammation after AcrySof Cachet implantation had a comparatively low peak after 1 week and returned to long-term safe levels at 1 month.
- No steroids seem to be needed in routine cases in addition to NSAIDS.
